# A Dynamic Deployment Method of Security Services Based on Malicious Behavior Knowledge Base

**DOI:** 10.3390/s22229021

**Published:** 2022-11-21

**Authors:** Qi Guo, Man Li, Weilin Wang, Ying Liu

**Affiliations:** School of Electronic and Information Engineering, Beijing Jiaotong University, Beijing 100044, China

**Keywords:** malicious behavior knowledge base, service function chain, security service, attack detection

## Abstract

In view of various security requirements, there are various security services in the network. In particular, DDoS attacks have various types and detection methods. How to flexibly combine security services and make full use of the information provided by security services have become urgent problems to be solved. This paper combines the reasoning ability of the malicious behavior knowledge base to realize the dynamic deployment of the service function chain and dynamic configuration of the security service function. The method feeds back the information generated by the security service to the knowledge base. After the analysis of the knowledge base, the service function chain path and the security service configuration policies are generated, and these policies will be dynamically distributed to the security service function. Finally, security services can be dynamically arranged for different network traffic, realizing the coordinated use of various security services and improving the overall detection rate of the network. The experimental results show that by arranging the paths under the UDP and the TCP, the overall detection rate of the network can reach 99% and 88%, respectively, indicating that it has a good overall detection performance for multiple distributed denial of service (DDoS) attacks.

## 1. Introduction

With the development of the Network Functions Virtualization (NFV), a large number of network function components based on special equipment have been flexibly deployed in the network in a software-based way [1], which provides the possibility for the flexible deployment of network services. Software-Defined Networking [2] (SDN) separates the control plane from the forwarding plane, and introduces programmability into the underlying network infrastructure, so that traffic can be processed in a more detailed and intelligent manner. NFV and SDN complement each other [3]. On this basis, the Service Function Chain [4] (SFC) brings new convenience to the flexible arrangement of network services and the provision of customized network services.

The service function chains based on SDN and NFV can flexibly arrange Virtual Network Functions (VNFs). By roughly classifying traffic, it can provide different service functions for different traffic. However, the service function chain relies more on the manual operation of the network administrator, and needs to manually arrange the service function path in advance, which brings limitations to the management of the network. Especially in terms of security services, in the face of a complex network environment, it is time-consuming and easy to increase the link load to provide a unified security service for all traffic [5]. The framework for Interface to Network Security Functions (I2NSFs) [6] provides an interface that maps the user requirements translation to Network Security Functions (NSFs) and implements the configuration of security service functions from top to bottom. However, this method can only meet the simple needs of users, and cannot expect users to be able to grasp the current state of the network, let alone make full use of and orchestrate the service functions in the network.

Therefore, in view of the characteristics of rapid changes in user security requirements and diverse security detection services, we propose a dynamic deployment method of security services based on the malicious behavior knowledge base [7]. This method is based on SDN and NFV technology, which turns network security services (mainly DDoS detection services) into VNFs that can be flexibly deployed, and obtains the ability to accurately control traffic paths by adding programmable routing control management [8]. Using the malicious behavior knowledge base, by collecting the capability information and processing result information of the security service function, the image of the network status is obtained to generate the network service management policy. Network service management policies include changing the path of specific packets and configuring the detection or interception of certain types of packets. By combining with the I2NSF framework, the policy is issued and configured into the network security service, to realize the purpose of dynamically deploying the security service according to the network status. Finally, we can dynamically select security services and service sequences to configure the network, and dynamically configure general service functions such as IDS and firewall functions. We also provide a method to optimize the detection of multiple types of attacks.

The contribution of this study can be listed as follows:

- Combine the malicious behavior knowledge base with the current detection scheme.
- Provide detection result feedback, and service path policy and configuration policy issuing capabilities by supplementing and defining various interfaces.
- Provide a combination of multiple DDoS detection methods.

The rest of the paper is organized as follows: Section 2 presents the related work, while the layered network structure and interface design are presented in Section 3 and Section 4, respectively. The experimental environment and results are presented in Section 5. Finally, Section 6 concludes this paper.

## 2. Related Works

On traditional networks, there are various network services, but most of these services are provided by dedicated devices, such as traffic cleaning units, firewalls, and Intrusion Detection Systems. In order to arrange these services in a certain order, the concept of the service function chain appeared. Due to the rigidity of traditional network deployment, which can only rely on fixed hardware systems, system upgrades and the information transfer between service functions are difficult, and the effect provided by the service function chain is greatly limited. With the development of the SDN and NFV in recent years, the service function chain has more room to play. SDN decouples the data plane from the control plane, adds programmability to the network, greatly improves the flexibility of the network, and makes flow control and flow table delivery easier. The development of NFV makes network services virtualized and removes the disadvantage that the hardware system is difficult to upgrade and control. The service function chains based on the SDN/NFV has obvious advantages in flexibility and automation. It can provide different services for different network traffic in a more targeted manner.

In terms of dynamic arrangement of the security service functions, for example, Lukas et al. [9] proposed to change the order of the security service functions chain (SSFC). They used the security function to report traffic information, and the function controller calculated the required order. When attack traffic exists, the order of SSFC can be dynamically adjusted, for example, the security service with the most types of attacks is placed first, and the effectiveness of adjusting the order of service functions is proved through experiments. Dwiardhika et al. [10] proposed to place security VNFs based on security levels to meet the security requirements of each service function chain and optimize the layout for secure virtual network functions. The security-level-based approach provides fine-grained security support but is not dynamic enough. Hoang et al. [11] used network function virtualization technology to evaluate the delay of deploying VNFs in the SFC and the protection capability of NFV in the face of DDoS attacks or web attacks. Zhai et al. [12] considered the security demand level and security level, and proposed a security and delay optimization SFC deployment method based on the Viterbi algorithm. However, this paper did not consider how to select services with short delay when security services are differentiated. Liu et al. [13] studied the problem of SFC service provisioning by taking the dynamic nature of user requests into consideration. SFC can be readjusted to adapt to the service requests’ dynamics for better user experience. However, users’ understanding of the network is always limited. Li et al. [14] used a two-stage intelligent detection model that can distinguish multiple DDoS attacks, but it lacks initiative.

In terms of DDoS detection, there are many detection methods, and most of them are against a single attack. She et al. [15] introduced a clustering-based HTTP flooding attack detection method. Gao et al. [16] found that five features are effective for detecting Distributed Reflection DoS (DRDoS) attacks, and proposed a method to detect DRDoS attacks. Wu et al. [17] proposed a method to detect Low-rate DoS (LDDoS) attacks in the SDN domain. We also need a way to flexibly integrate these modules.

The I2NSF working group has currently formed three RFC standards in terms of issuing service function configuration information. RFC 8192 [18] addresses the current challenges faced by security service providers, gives a problem statement about I2NSF, and gives some supporting use cases. RFC 8329 [19] describes the architecture of I2NSF in detail and introduces the proposed interface. RFC 9061 [20] describes the issue of providing IPsec-based traffic protection (integrity and confidentiality) over network interfaces, allowing the I2NSF controller to configure and monitor network security functions. Another draft [21] describes how to integrate the I2NSF framework into the NFV reference model. This draft also provides a use case that uses an SFC-enabled I2NSF. The draft [22] describes an extension of the I2NSF framework for security automation management under cloud-based security services.

## 3. Dynamic Deployment Method of Security Service Function

We propose a layered network structure based on the I2NSF framework and SDN, and divide the network into three layers according to their functions. In Section 3.1, we introduce the overall network framework. In Section 3.2, we introduce the upper layer that dynamically generates network service management policies based on the bottom layer processing information. In Section 3.3, we introduce the middle layer, which implements the translation of policies and the role of distribution configuration. It enables the network to dynamically combine security service functions according to the network state. In Section 3.4, we introduce the bottom layer that implements traffic processing and information collection.

### 3.1. Security Service Dynamic Deployment Framework

**Figure 1:**
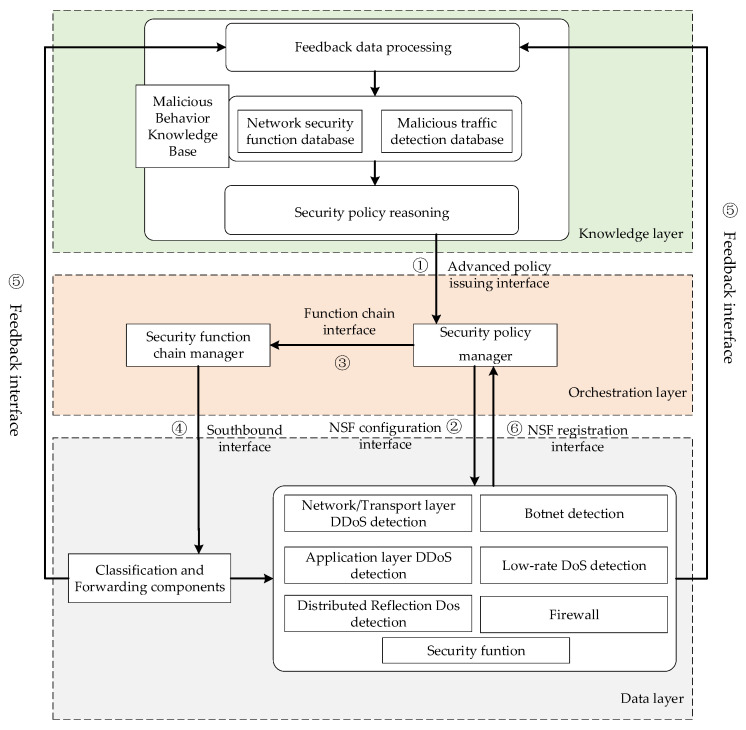
Block diagram of dynamic deployment of security services.

In order to realize the dynamic deployment of network security functions, a dynamic deployment model of network security functions, as shown in Figure 1, is proposed. It divides the network into three layers, namely the knowledge layer, the orchestration layer, and the data layer, and defines six types of interfaces, namely the advanced policy issuing interface, NSF registration interface, NSF configuration interface, function chain interface, southbound interface, and feedback interface. We use the YANG model to define standardized interfaces, and we connect the underlying network with the upper-level malicious behavior knowledge base. We use security services to process traffic, and dynamically arrange and configure security services.

### 3.2. The Knowledge Layer

**Figure 2:**
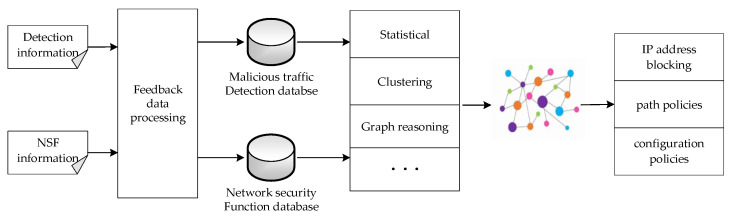
Processing flow of malicious behavior knowledge base block diagram.

As the top layer of the dynamic deployment of security services, the knowledge layer takes the malicious behavior knowledge base [7] as the core. It stores network security function information and security function detection results, including a feedback data processing module, a repository, and a security policy reasoning module. As shown in Figure 2, the knowledge layer establishes a malicious traffic detection database by collecting data feedback of security functions, and establishes a security function database by collecting network security function information. It dynamically generates advanced security policies through the security policy reasoning module. The security policy is finally issued to the orchestration layer through the advanced policy issuing interface for further processing.

The feedback data processing module receives the feedback data from the data layer, performs structured processing on the received data according to the data model of the knowledge base, and stores the data in different knowledge bases. The network security function database stores the description of network security functions and corresponding security capabilities in the data layer. For example, five attack detection modules are used as detection security functions in this experiment, and there are also security functions such as the Intrusion Detection System (IDS) and firewall. The malicious traffic detection database stores the malicious traffic records detected by the detection module; based on the knowledge graph, the malicious behavior knowledge base is constructed for the stored information in the form of entities, relationships, and attributes, and each entity or relationship may have different multiple attributes.

According to the stored information, the security policy reasoning module can use statistical analysis, the clustering algorithm, the graph reasoning algorithm, and other methods to realize malicious behavior perception and classification. It can generate IP-address-blocking policies for IP addresses judged to be malicious, and optimize path policies and configuration policies for network conditions.

### 3.3. Orchestration Layer

**Figure 3:**
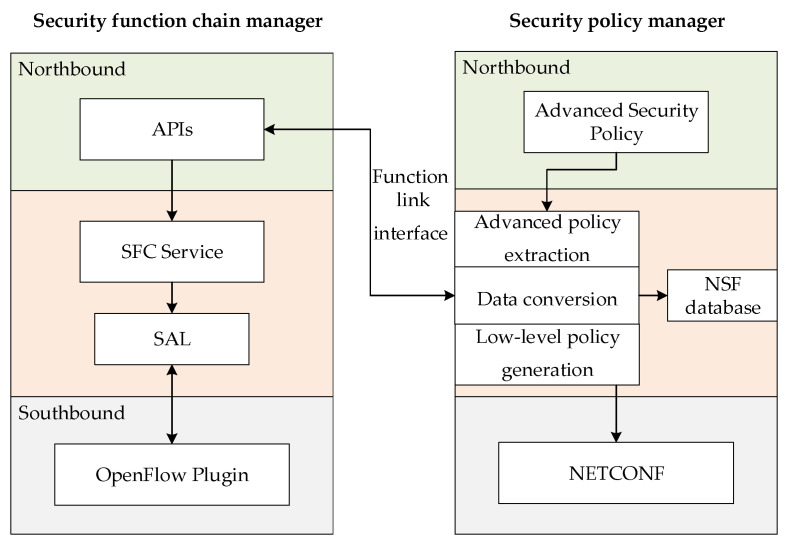
Orchestration Layer Module.

The orchestration layer consists of a security function chain manager and a security policy manager as shown in Figure 3. It is used to translate the high-level policy, determine the service function that can execute the policy, and implement path distribution and low-level policy configuration.

The security policy manager contains the NSF database, advanced policy extraction module, data conversion module, and low-level policy generation module. The NSF database stores the location information of the NSF and endpoint group, such as the IP address of the service function forwarder (SFF) where the NSF is located and its own address. The advanced policy extraction module is based on finite state automata, which can recognize the input characters, make state transitions, and obtain the final state. The data conversion module will select an NSF that can execute the high-level policy, and convert the advanced policy into a low-level policy executable on a certain NSF. The low-level policy generation module will use the location database to translate endpoint information and NSF names into specific IP addresses.

As a special NSF, the security function chain manager receives the path policy information from the security policy manager, converts it into a flow table, and sends it to the classifier forwarder (CF) and SFF. The security function chain manager has an SFC service module, service abstraction layer (SAL) module, and southbound interface. In this paper, OpenDaylight is used as the security function chain manager, and the SFC component is added to facilitate the connection with the data layer to realize the service function chain. The classification criteria in the path policy are combined with the path information to jointly determine the flow table information on the CF and SFF.

The interactive process between the control layer modules is as follows:

1. The security policy manager monitors the advanced security policies issued by the knowledge layer;
2. The security policy manager uses the advanced policy extraction module to extract the information in the advanced security policy based on the deterministic finite automaton (DFA);
3. The security policy manager uses the data conversion module to further convert the advanced security policy. It converts abstract information into concrete execution information on the NSF;
4. The security policy manager uses the low-level policy generation module to generate a low-level security policy in the format required by the NSF configuration interface, sends the low-level path policy to the function link interface, and sends the low-level configuration policy to the southbound NETCONF [23] interface;
5. The NETCONF interface of the security policy manager establishes a NETCONF connection with the corresponding NSF, and sends the low-level security policy;
6. The security function chain manager generates a configuration file for the received low-level path policy information, and sends it to the SDN switch through the SAL module using the OpenFlow [24] protocol.

### 3.4. Data Layer

**Figure 4:**
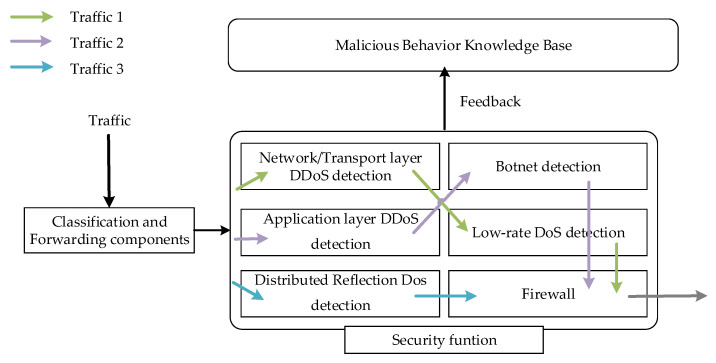
Data layer module.

The data layer is the bottom layer for receiving configuration information, implementing traffic forwarding and various security services. It consists of classification and forwarding components, various malicious traffic detection modules, and various security service modules, as shown in Figure 4. After the network traffic is classified, it is processed in different modules.

The classification and forwarding components are the CF and SFF in the SFC framework, corresponding to the switches in the SDN network, and can use the flow table to forward traffic. This paper uses Open vSwitch (OVS) and adds the Network Service Header (NSH) [25] patch to implement this component. OVS is an open-source software switch that can be installed in a general-purpose virtual server environment. The NSH provides a common standard header and implements an Overlay network, which uses logical links on the existing network to form a virtual network.

The NSH consists of a 4-byte Base Header, a 4-byte Service Path Header, and a Context Header of optional length. The Service Path Header provides path identification information and path location information. It uniquely identifies the path and the position in the path in the data packet. This paper uses the Layer 2 frame as the upper layer protocol of NSH and adopts VxLAN-GPE as the transmission protocol.

The attack detection module can perform attack detection on the traffic passing through the module, and can upload the detection results to the malicious behavior knowledge base through the feedback interface. This paper uses the Network/Transport layer DDoS (NetDDoS) detection module [14], Low-rate DoS (LDDoS) detection module [26], Botnet detection module [27], Application layer DDoS (AppDDoS) detection module [28], and Distributed Reflection Dos (DRDoS) detection module [29]. Through different combinations of these five detection modules, efficient DDoS attack detection effects are achieved. In addition, the data layer also has functional modules such as the IDS and firewall, which jointly provide services for traffic.

## 4. Interface Design

We design six types of interfaces for realizing the dynamic deployment of security services. In Section 4.1, we introduce an advanced policy issuing interface, which issues the path policy and configuration policy generated by the malicious knowledge base. In Section 4.2, we introduce the NSF configuration interface and function link interface. They are used to issue low-level policies to the NSF. In Section 4.3, we introduce the feedback interface and NSF registration interface. They are used to register the location information of the NSF and send back detection results to the knowledge base. Through these interfaces, we realize the path policy and configuration policy issued by the malicious knowledge base, the security service function feeds back the processing result information, and the security service function returns the registration information.

### 4.1. Advanced Policy Issuing Interface

The advanced policy issuing interface defines the information model and data model for issuing advanced security policies from the knowledge layer to the orchestration layer. Advanced security policies in this article include advanced configuration policies and advanced path policies. The advanced configuration policy is formed on the basis of the advanced policy in the I2NSF framework [30], and the advanced path policy is given in combination with the service function chain. These two types of policies allow specific traffic to pass only the security function it needs and enable a custom configuration of that security function.

**Figure 5:**
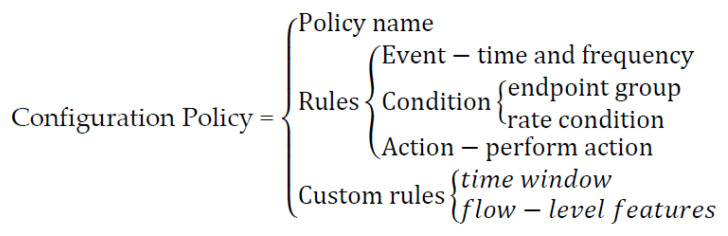
Advanced Configuration Policy.

The advanced configuration policy is the specific configuration of different security functions, such as the configuration policy for the firewall and the configuration policy for the IDS. Configuration policies include policy names, rule sets, endpoint groups, and custom rules, as shown in Figure 5. The rule set consists of an event–condition–action model. Each configuration policy may have multiple rule sets.

A rule contains the rule name, event, condition, and action. Events mainly define time and frequency information. Conditions contain inspection conditions that apply to target traffic. Actions indicate what actions should be performed when a rule is matched.

Action objects include operations such as pass, drop, reject, rate limit, and forward. Endpoint groups are used in policies to specify the endpoints for which they are intended to be used. Endpoint groups include user groups, device groups, location groups, and URL groups. Each group includes information such as the given IPv4 address, IPv6 address, MAC address, or URL address. For example, the user group includes information about the user’s MAC address and IP address or IP address range.

Custom rules are self-defined configuration policies based on NSF’s functional information. In this paper, two custom rules are designed mainly for the characteristics of the detection module, which are the time window of the detection module and the flow-level features required for detection.

**Figure 6:**
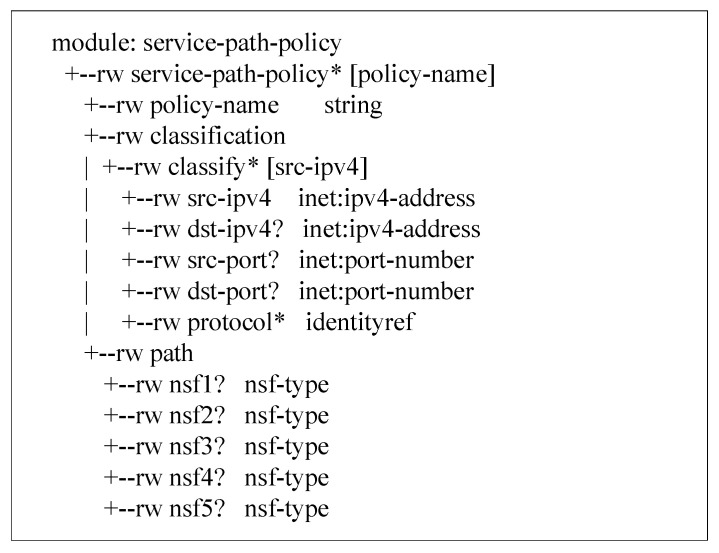
Advanced Path Policy Information Model.

The path policy mainly includes two parts, the classification policy and security function selection policy. The classification policy classifies packets by source IP address, destination IP address, source port, destination port, and protocol type. The security function uses the five types of attack detection modules proposed in the current experiment and the Firewall and IDS as options. The path policy data model information is shown in Figure 6. (The options with asterisks in the figure are mandatory.) The classification policy and the security function selection policy together determine that a packet should enter the required security functions in a certain order. The data model of the path policy is shown in Table 1.

### 4.2. NSF Configuration Interface and Function Link Interface

In the NSF configuration interface, this paper mainly uses the information model and data model defined in the draft [31] to configure the service function through the NETCONF interface. The NSF-oriented data model is generated by the security policy manager through the translation and transformation of advanced security policies. Therefore, the data model does not have more information than the high-level policy but becomes the specific configuration information. For example, the endpoint group in the rule is converted into specific location information, and the matching NSF is selected for the action.

**Figure 7:**
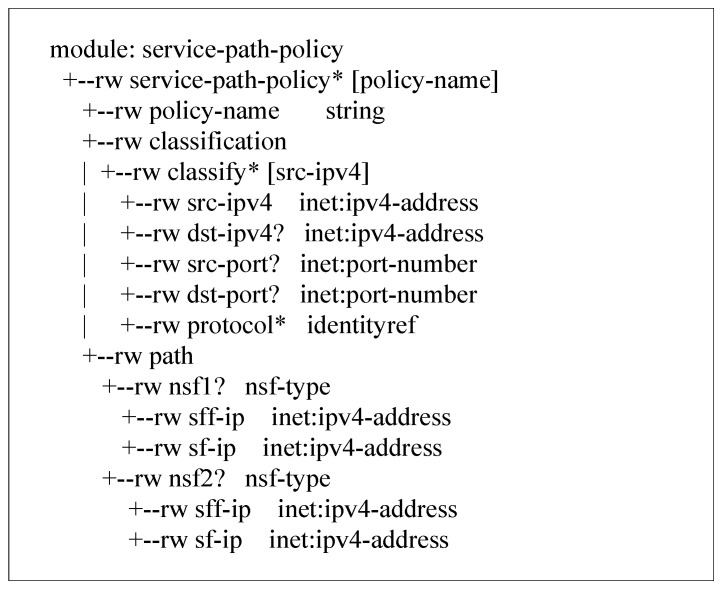
Information Model for Low-Level Path Policy.

The function chain interface mainly delivers the path policy in the high-level security policy and sends the low-level path policy information as shown in Figure 7 to the security function chain manager. After receiving the advanced path policy, the security policy manager maps the service function name to a specific IP address and sends it to the security function chain manager through the function chain interface. Further configuration is performed by the security function chain manager. The security function chain manager uses the OpenFlow protocol to configure the classification policy and path on the data layer to realize the service function chain.

### 4.3. Feedback Interface and NSF Registration Interface

The function of the feedback interface is to send the security capabilities and detection results of the NSF to the malicious behavior knowledge base. The description of the security capabilities of the NSF determines which type of traffic can be used for security detection by the knowledge layer. The results of the security detection report the current state of the network to the knowledge layer. This paper defines eight security functions, and the basic information is described as follows, including the security function name, packet level/flow level, function classification, and function description. The security detection results provide different flow-level features and detection result information for five different security detection models.

**Figure 8:**
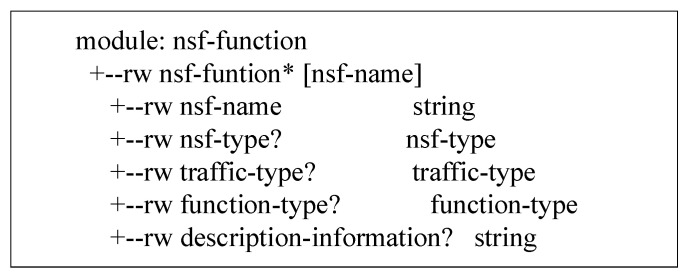
Information Model for Feedback of NSF Capability.

The YANG information model of the feedback service function capability information defined in this paper is shown in Figure 8. The information model defines the basic structure of feedback information, including service function name, type, traffic type, function type, and capability description information. The specific data model classification is shown in Table 2.

The feedback interface also feeds back the detection result of the malicious traffic detection module to the knowledge layer. The YANG information model of the feedback interface is defined as follows, including the detection module name, service function type, traffic quintuple information, flow-level features at the statistical level, and detection results. The data model definition of the detection results of the malicious traffic detection module is shown in Table 3.

**Figure 9:**
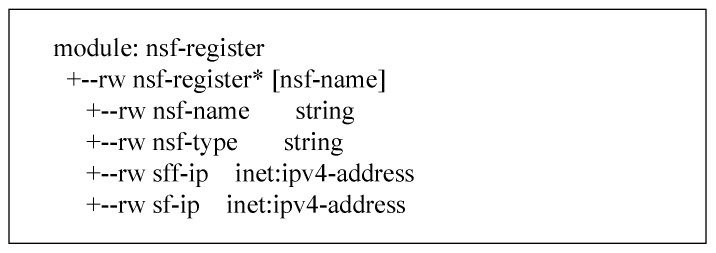
The information model of the registration interface.

The NSF registration interface as shown in Figure 9 sends the location information of the NSF and the IP address information of the SFF where it is located to the security policy manager. The Security Policy Manager stores and updates the latest service function location information and provides data information for translating advanced configuration policies. The registration interface information model is as follows, including the NSF name, NSF type, IP address of the SFF, and its own IP address.

## 5. Experimental Results

This section introduces a prototype system for a dynamic deployment method of security services based on the malicious behavior knowledge base. We replay various DDoS attack traffic, simulate normal communication requests in the 5G environment, and orchestrate security service functions with targeted DDoS detection capabilities. In Section 5.1, we introduce the experimental environment and experimental program. In Section 5.2, we propose some new evaluation metrics to evaluate the detection rate. In Section 5.3, we present the results of the experiment. It is verified that the dynamic deployment method of security services proposed in this paper can fully utilize the functional capabilities of security services and provide detection of various types of DDoS attacks.

### 5.1. Experimental Program

**Figure 10:**
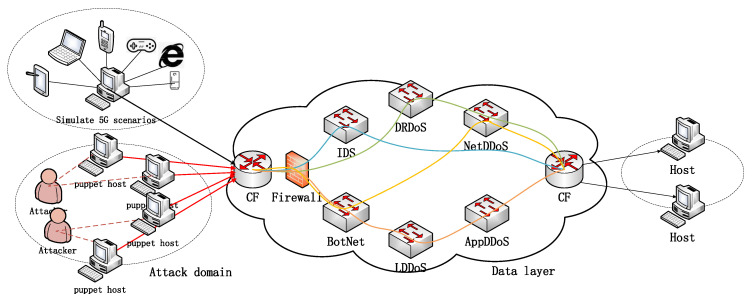
Data Layer Security Function Dynamic Deployment Experimental Topology.

An experimental environment is built based on VMware vSphere, and the experimental topology is shown in Figure 10. The configuration of each virtual machine is 16 GB of memory and 30 GB of hard disk space. A total of 11 hosts are used in the experiment. It includes a security service chain manager, a security policy manager, two classifiers, four forwarders, a knowledge layer host, a host for sending attack traffic and normal traffic, and a host for receiving. The security service chain manager adopts OpenDaylight and adds the SFC component, and the classifier and forwarder use Open vSwitch and add the NSH [25] patch. OpenDaylight [32] is the most widely deployed open-source SDN controller platform. Open vSwitch is a production-quality, multilayer virtual switch. Different service functions are installed on the SFF, and Docker containers are used to isolate various functions. The SFC proxy script is used in the container to help match and change the NSH information.

In order to more comprehensively compare the detection effects of various DDoS attacks, this paper uses Tcpreplay to replay five types of DDoS attacks, including Distributed Reflection Denial-of-Service, Network/Transport layer DDoS, Application layer DDoS, Botnet, and Low-rate Denial-of-Service attacks. There are multiple subtypes of attacks under each type of attack, as shown in Table 4.

The simulated 5G scenario replays the massive normal communication data requests generated in the paper [33] under the four scenarios of simulated public service, smart home, PC internet access, and massive Machine Type Communication (mMTC).

### 5.2. Evaluation Metrics

In order to make full use of the detection results of multiple modules on traffic, this paper uses three detection indicators to evaluate the detection effect, namely the accuracy rate, the malicious traffic reduction rate, and the comprehensive detection rate. The accuracy rate represents the proportion of the number of correct samples classified by the model to the total number of samples. The malicious traffic reduction rate represents the proportion of the detected malicious traffic to the total malicious traffic, and the calculation formula is shown in Equation (1). The comprehensive detection rate is the synthesis of each detection module. First, the concept of stream ID is defined. The source IP address, source port number, destination IP address, destination port number, and protocol type form the flow ID. When sending an attack, the flow ID of each type of attack is unique, and the attack label is also generated according to the flow ID. Therefore, the concept of the host is replaced by the flow ID. Each module may have multiple detection results for a flow ID, and the detection results accounting for more than 2/3 of them are selected as the judgment results of the module. When each module achieves a unified result, the final result is obtained. The comprehensive detection rate is the ratio of the number of correct flow IDs in the final result to the total number of flow IDs, indicating the detection rate of a host. The calculation formula is shown in Equation (2).

(1) Malicious traffic detection rate=1−∑P/∑N

(2) Comprehensive detection rate=∑T/∑A

Among them, *P* is the data samples that have not been detected in the online detection, and *N* is all the attack data samples. *T* is the number of categories of flow IDs whose detection results are correct, and *A* is the total number of flow ID categories.

### 5.3. Experimental Results

The path policy in the experiment is very flexible. You can set various path policies according to IP address, port number, protocol number, etc. In Section 5.3.1 and Section 5.3.2, we use a typical large category classification method, which shows that under the condition of insufficient knowledge of network information, the classification detection based on the type of transport layer protocol is also effective. In Section 5.3.3, a more detailed policy is used, which shows that we can use multiple path polices and blocking polices dynamically.

#### 5.3.1. Path Policy and Configuration Policy Delivery

To issue path policies and configuration policies, you first need to register various types of information, such as address information of service functions and condition information in advanced policies. The address information of the service function mainly includes the IP address of the SFF and the Docker container address of the service function. The condition information in the advanced policy mainly registers the address information of the port. Table 5 shows the result of registering the service function, which realizes the mapping of service function name and location information. Table 6 shows the result of registering users and devices. These addresses correspond to endpoint groups in the rule set.

**Figure 11:**
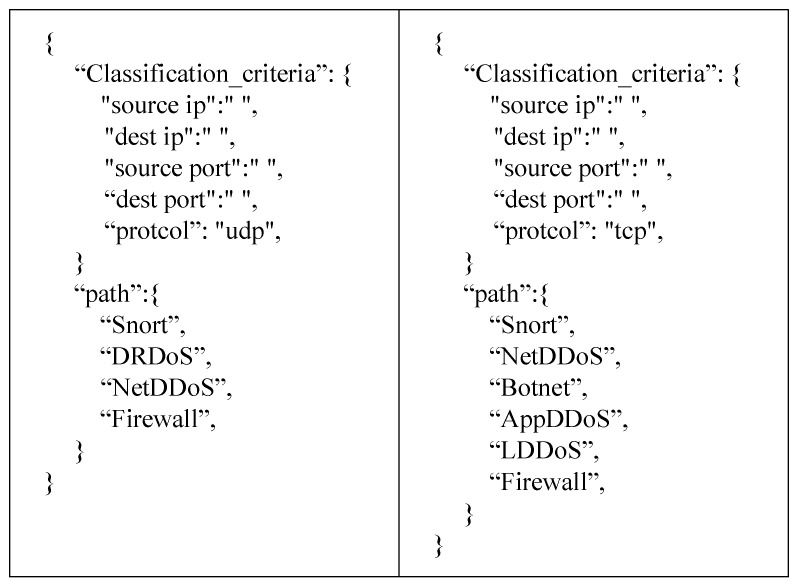
Path policies under UDP and TCP.

After completing the above information registration, the security policy manager can obtain the NSF location information by reading the NSF registry. The knowledge layer can issue path policies to realize the configuration of the service function chain. As shown in Figure 11, two path policies as examples are used to implement different service functions for TCP and UDP packets. The UDP packets will pass through the four service functions of Snort, the firewall, the DRDoS Detection module, and the NetDDoS Detection module. The TCP packets will pass through the six service functions of Snort, the firewall, the Botnet Detection module, the AppDDoS Detection module, the LDDoS Detection module, and the NetDDoS Detection module. We examine the detection rate and latency under these two policies.

**Figure 12:**
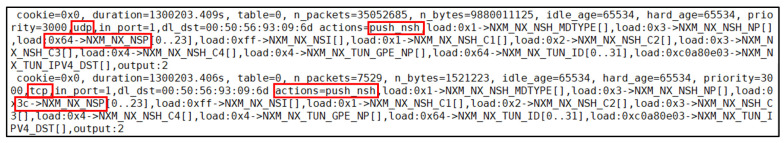
Classifier flow table.

The flow table of the path policy on the classifier is shown in Figure 12. Red box indicates that different NSPs are added to UDP and TCP traffic, and that different paths are used. Different paths have different NSP information. Packets of UDPs and TCPs are added to NSHs with different path identifiers.

**Figure 13:**
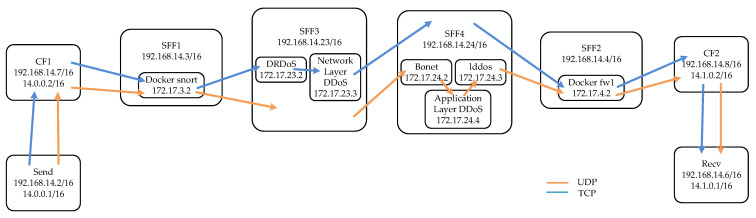
UDPs path and TCPs path.

Finally, the service function chain path for UDP packet traffic, as shown in Figure 13, is implemented at the data layer. The UDP packet will pass through CF1 in order to add the NSH, then pass through SFF1, SFF3, and SFF2 in turn to obtain four types of services, and finally through CF2 to restore the original message. The blue line in Figure 13 shows the service function chain path for TCPs traffic. TCPs pass through Snort, the firewall, and sff1, sff3, sff4, and sff2 to obtain six network services.

#### 5.3.2. Statistical Results Analysis

The detection results in the malicious behavior knowledge base are summarized, and the detection results of each module are shown in Table 7.

From the detection results, we can see that in the case of binary classification, when we only need to distinguish malicious traffic, the detection results are very good. The accuracy rate is mostly above 90%, and the malicious traffic detection rate is also close to 1, indicating that most malicious traffic is distinguished. However, in the multiclass case, the detection results are poor. The reason is that different modules also detect other kinds of malicious traffic, and it is easy to misclassify the attack traffic of other modules. Table 8 shows the comprehensive detection rate and the number of detected flow IDs under the two paths. The comprehensive detection rate under the UDP path is 99% and it is 88% under the TCP path. Due to the poor multiclassification results of the TCP itself, the comprehensive detection result reaches 88%, which shows that this method can improve the overall detection rate on the basis of using the original detection algorithm. Thus, under the above two paths, despite our most stringent multiclassification criteria, the results are still good. This is attributed to the comprehensive utilization of the detection results of multiple modules by the malicious behavior knowledge base.

**Figure 14:**
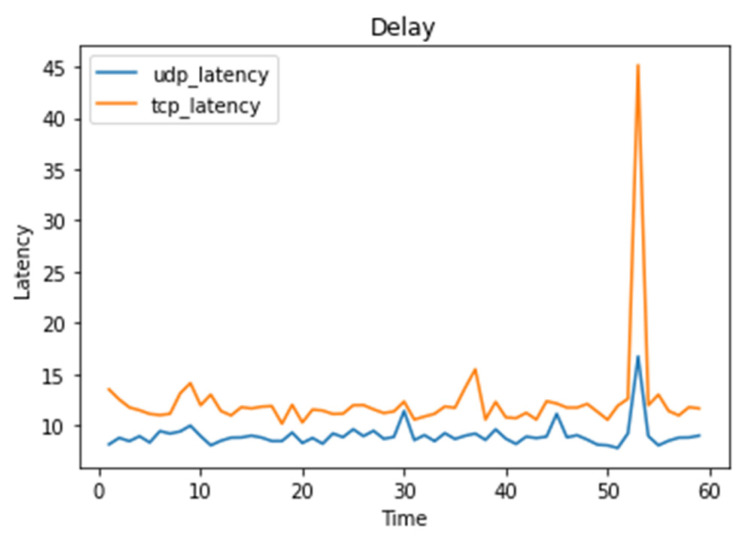
Path Delay Comparison.

When selecting different paths, the number of service functions passed is different. As shown in Figure 14, using the Iperf tool, we compare the delay time under the UDP path and TCP path within one minute.

Under the UDP path, traffic passes through four service functions, namely Snort, the DRDoS detection module, the NetDDoS detection module, and the firewall, with an average delay of 9.04 milliseconds. Under the TCP path, traffic passes through six service functions, namely Snort, the NetDDoS detection module, the Botnet detection module, the AppDDoS detection module, the LDDoS detection module, and the firewall, with an average delay of 12.33 milliseconds. It can be seen that, by simply classifying the protocol types, the time for providing security services can be greatly saved. The latency peak comes from network blocking when the traffic is too large, and because the experiment is based on the virtual switch, the performance of the virtual switch is not stable enough, and there may be disturbances in the execution process. In addition, because a packet may pass through multiple security services, in terms of large-scale, high-rate attacks, it may cause more network congestion.

#### 5.3.3. Dynamic Adjustment Policy

According to the detection results or user requirements, we can dynamically change the path policy and configuration policy. When multiple types of network attacks pass through, we can implement specific path policies for specific hosts. The blocking policy can be implemented for hosts with malicious detection results. For user requirements, we can also configure them on the network, and this dynamic distribution policy is combined with the malicious behavior knowledge base.

**Figure 15:**
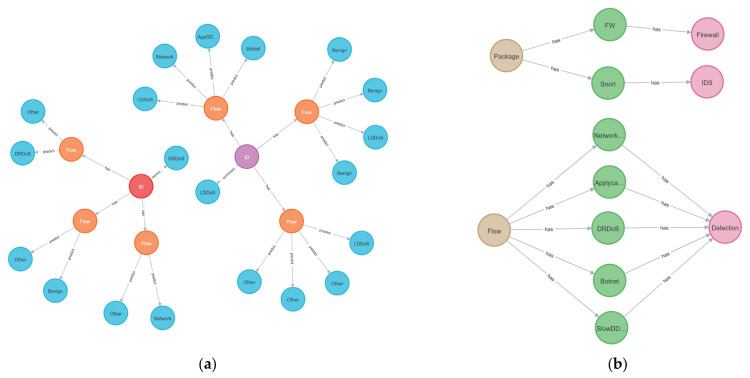
Data display of malicious behavior knowledge base. (**a**) Malicious traffic detection graph. (**b**) NSF Capability Graph.

The malicious behavior knowledge base uses the NSF capability information and detection results to build a knowledge graph based on the Neo4j graph database to visualize the data model. It displays the structure and link relationship of data in the form of a node link graph, forming a malicious traffic detection graph, as shown in Figure 15a, and an NSF capability graph, as shown in Figure 15b. The malicious traffic detection graph shows that there are multiple flows with the same flow ID, each with multiple detections. By combining multiple detections and multiple flows, it is possible to predict whether a flow ID is malicious or not. The NSF capability graph mainly describes the type of NSF, which can help the knowledge base better understand the role of each service function.

**Figure 16:**
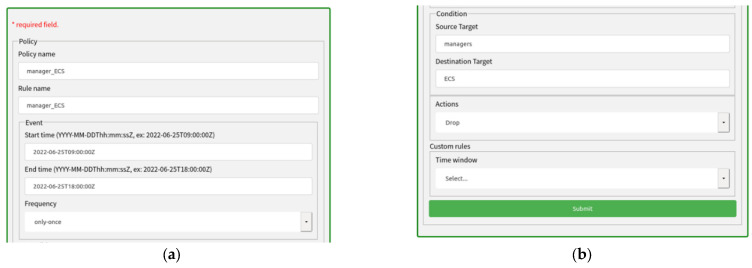
Advanced policy for blocking traffic. (**a**) Policy name and event. (**b**) Condition, actions and custom rules.

Based on the knowledge inference of the malicious behavior knowledge base, path policies and configuration policies can be easily and dynamically adjusted, and more detailed paths and configurations can be used. Figure 16 shows that the manager user is blocked from accessing Elastic Compute Service (ECS) resources between 9:00 and 18:00. The addresses of the manager and ECS are registered in the NSF database in advance.

**Figure 17:**
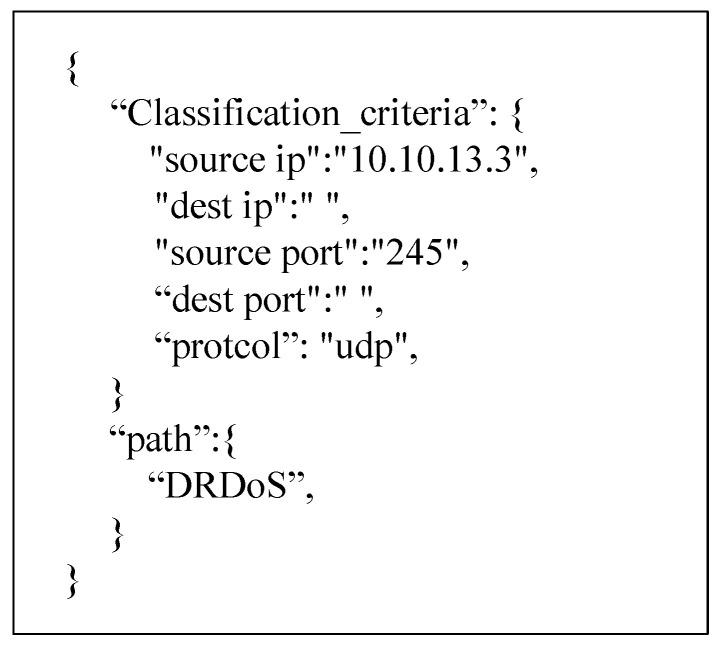
Dynamically adjusting the path policy.

Further path policy refinement can also be performed according to the characteristics of the service function. For example, for a certain IP address, only one detection module can be used to shorten the service time. The path policy is shown in Figure 17. The policy indicates that the traffic under the UDP whose source IP address is 10.10.13.3 and source port number is 245 will only pass the DRDoS Detection module to complete the detection.

For the AppDDoS service function and the LDDoS service function, compared with other service functions, these two types of functions focus more on detecting HTTP traffic. HTTP packets use port number 80. Therefore, to detect DDoS attacks related to the HTTP, packets with port number 80 can be detected using only these two service functions.

## 6. Conclusions

We propose a dynamic deployment method for security services based on a malicious behavior knowledge base. Its network architecture is divided into three layers and six interfaces. The dynamic distribution of service function paths and security service configurations is realized. It is able to provide dynamically adjusted security services based on the different packets in the network, choreographing multiple detection modules to improve the overall network detection rate and reduce detection latency. It makes full use of the processing information of security services and establishes a mechanism that can feedback processing information and dynamically configure the network. The network can be dynamically configured, mainly by selecting security services and service sequences, and general service functions can be configured, such as IDS and firewall functions. In addition, in the aspect of DDoS attack detection, it provides a method to optimize the detection of multiple types of attacks. The experimental results show that the overall detection rate of the combined multimodule detection for multiple types of attacks is over 88%. Further research will follow on dynamically tuned services, as well as on the moving target defense.

## Figures and Tables

**Table 1:** Advanced Path Policy Data Model.

Path Policy	
Classification criteria	source IP address
	destination IP address
	source port
	destination port
	protocol type
path information	NSF name

**Table 2:** Path Policy Data Model.

NSF-Type	Traffic-Type	Function-Type	Description Information
DRDoS	Flow	DDoS Detection	Detect DRDoS Attack
Network	Flow	DDoS Detection	Detect Network/Transport layer DDoS Attack
Botnet	Flow	DDoS Detection	Detect Botnet Attack
AppDDoS	Flow	DDoS Detection	Detect Application layer DDoS Attack
LDDoS	Flow	DDoS Detection	Detect Low-rate DDoS Attack
Firewall	Package	Firewall	Firewall
URL-Filter	Package	Filter URLs	Filter packets based on URL
Snort	Package	IDS	Intrusion Detection System
Suricata	Package	IDS/IPS	Intrusion Detection System/Intrusion Protection System

**Table 3:** Detection result table of each module.

Detection Module Name	Traffic Quintuple Features	Flow-Level Features	Detection Result
DRDoS	IP, Port, Protocol	S_time, E_time, etc.	Benign, etc.
Network	IP, Port, Protocol	Average Packet Size, etc.	SYN Flood, etc.
Botnet	IP, Port, Protocol	SYN Flag Count, etc.	Ares, etc.
AppDDoS	IP, Port, Protocol	Flow Duration, etc.	HTTP Flood, etc.
LDDoS	IP, Port, Protocol	FIN Flag Count, etc.	Slow Read, etc.

**Table 4:** Type of attack.

Type of Attack	Attack Subclass					
DRDoS	Chargen	NTP	SSDP	TFTP		
LDDoS	Slow Headers	Slow Body	Slow Read	Shrew		
AppDDoS	CC	HTTP Flood	HTTP Get	HTTP Post		
Network	UDP-Flood	ACK-Flood	SYN-Flood			
Botnet	Shrew	Mirai	BYOB	Ares	IRC-Botnet	Zeus

**Table 5:** Service Function Registry Information.

Name	sff_ip	sf_ip
DRDoS	192.168.14.23	172.17.23.1
NetDDoS	192.168.14.23	172.17.23.2
Botnet	192.168.14.24	172.17.24.2
AppDDoS	192.168.14.24	172.17.24.4
LDDoS	192.168.14.24	172.17.24.3
Snort	192.168.14.3	172.17.3.2
Firewall	192.168.14.4	172.17.4.2

**Table 6:** Device Address Registry Information.

Name	start_ip	end_ip
ECS	10.1.0.1	10.1.0.20
Manager	14.0.0.1	14.0.0.10

**Table 7:** The detection results of each module.

	DRDoS	NetDDoS	AppDDoS	BotNet	LDDoS
Binary classification accuracy	0.9997	0.9119	0.9993	0.9535	0.7138
Malicious traffic detection rate	1.0	0.996	1.0	1.0	0.769
Multiclass Accuracy	0.9997	0.696	0.943	0.629	0.438

**Table 8:** Comprehensive detection rate.

	UDP Path	TCP Path
Comprehensive detection rate	0.9996	0.8885
Total number of flow IDs	2856	2441

## Data Availability

The dataset created in this article can be found on github: https://github.com/liliMpro/source_dataset (accessed on 12 October 2022).
